# Mesenchymal stem cells derived from adipose accelerate the progression of colon cancer by inducing a MT-CAFs phenotype via TRPC3/NF-KB axis

**DOI:** 10.1186/s13287-022-03017-5

**Published:** 2022-07-23

**Authors:** Chunling Xue, Yang Gao, Xuechun Li, Mingjia Zhang, Ying Yang, Qin Han, Zhao Sun, Chunmei Bai, Robert Chunhua Zhao

**Affiliations:** 1 Beijing Key Laboratory (No.BZO38 1), Center of Excellence in Tissue Engineering Chinese Academy of Medical Sciences, School of Basic Medicine Peking Union Medical College, Institute of Basic Medical Sciences Chinese Academy of Medical Sciences, Peking Union Medical College Hospital, No. 1 Shuaifuyuan Hutong, Dongcheng District, Beijing, 100730 People’s Republic of China; 2 Department of Oncology, Peking Union Medical College Hospital, Chinese Academy of Medical Science and Peking Union Medical College, No. 1 Shuaifuyuan Hutong, Dongcheng District, Beijing, 100730 People’s Republic of China

**Keywords:** Exosomes, Colon cancer, Growth and metastasis, TRPC3, NF-KB signaling pathway, Prognosis

## Abstract

**Background:**

There is increasing evidence that mesenchymal stem cells (MSCs) help shape the tumor microenvironment and promote tumor progression, and ion channels might play a critical role in this process. The objective of the present study was to explore the function and mechanism of MT-CAFs on progression of colon cancer.

**Methods:**

Here, a gene chip was used for a general analysis of gene expression changes in MSC-transformed CAF cells (MT-CAFs). Bioinformatic tool and western blot screened out the ion channel protein TRPC3 with significantly increased expression, and identify the function through two-photon microscope. The progression of cancer was detected via MTS, transwell and Wound Healing. ELISA deected the secretion of inflammation factors. TRPC3/NF-KB axis was identified by western blot and immunofluorescence.

**Results:**

TRPC3 can caused calcium influx, which further activated the NF-KB signaling pathway. Knockdown or inhibition of TRPC3 in MSCs significantly reduced the activation of NF-KB, and decreased the growth, migration, and invasion of MT-CAFs. After TRPC3 knockdown, the ability of MT- CAFs to promote tumor migration and invasion was impaired. Conversely, the upregulation of TRPC3 expression in MT-CAFs had the opposite effect. In vivo, TRPC3 expressed on MSCs also contributed to the tumorigenesis and progression of cancer cells. In addition, the Oncomine and GEPIA databases showed that TRPC3 expression is higher in colon cancer tissues compared with normal colon tissues, and was positively correlated with the expression of the CAF genes alpha-smooth muscle (*α*-SMA/ACTA2) and fibroblast activation protein Alpha. The disease-free survival of patients with positive TRPC3 expression in MSCs was significantly shorter than those with negative expression.

**Conclusions:**

These results indicate that TRPC3 expressed on MT-CAFs plays a critical role in tumor progression via the NF-KB signaling pathway, and is correlated with poor prognosis in colon cancer patients. Therefore, TRPC3 may be a novel therapeutic target for the treatment of colon cancer.

**Supplementary Information:**

The online version contains supplementary material available at 10.1186/s13287-022-03017-5.

## Introduction

Colorectal cancer is one of the leading causes of cancer-related mortality [1, 2]. The progression of cancer is positively related to intratumoral heterogeneity such as genetic mutations and changes in the local tissue microenvironment [3]. The colorectal cancer niche contains various kinds of cells, including immune cells, inflammatory cells, intratumoral macrophages and fibroblasts and so on, which can regulate the progression of cancer by secreting proinflammatory factors [4]. Consequently, there is a complex and mutual effect between many different cellular and acellular elements in the tumor microenvironment [5].

Mesenchymal cells of the intestines have specific features and molecular markers that offer mechanical support and play a critical role in the process of intestinal morphogenesis [6, 7]. Moreover, there are many reports that mesenchymal stem cells (MSCs) have the ability to transform into cancer associated fibroblasts (CAFs) in the tumor microenvironment, and this process has become an important field in oncology [8, 9]. Colorectal tumor cells secrete many factors, including inflammatory factors, immunomodulatory factors, chemokines and so on, which can affect the biological behavior of MSCs [10, 11]. However, the interactions between MSCs and colon cancer cells remains incompletely understood.There is increasing evidence that different ion channels play an important role in various human cancers [12], modulating the migration, invasion and proliferation of cancer cells [13, 14]. Ca^2+^ influx plays a critical role in the function and fate of many different cell types, and TRPC3 is known to regulate Ca^2+^ flux by a nonselective permeation pathway [15]. TRPC3 presents in the plasma membrane and mitochondrial inner membrane [16, 17]. As a regulator, TRPC3 was reported to promote the migration and invasion of several different tumor cell types [12]. For example, TRPC3 channel promotes the growth of human ovarian cancer cells [12, 18], and it can modulate the growth and apoptosis resistance of triple negative breast cancer cells via the TRPC3/RASA4/MAPK signaling pathway [19]. Moreover, LncRNA SNHG5 plays a critical role in the growth and invasion process of melanoma via the miR-26a-5p/TRPC3 signaling pathway [20]. However, the effect of TRPC3 expressed on MT-CAFs in the tumor microenvironment has not been reported.

In the present study, we emphasize the crucial role of TRPC3 channel in the invasion and migration of MT-CAFs and its promoting effect on colon cancer cells. The results indicate that TRPC3 may be a potential new therapeutic target for the treatment of colorectal cancer.

## Materials and methods

### Cell culture

The HCT116 cells were cultured as described in the literature [21].

### Isolation and culture of human adipose-derived MSCs (hAD-MSCs)

We collected adult fat samples from Peking Union Medical College Hospital (PUMCH) after acquiring informed consent from the patients. The adipose tissue was washed three times using D-Hanks’ buffer with penicillin (100 IU)/streptom- ycin(100ug/ml), and then centrifuged at 800 g for 3 min. The supernatant was transferred into a fresh 50 ml centrifuge tube. And the pelleted tissue was treated with 0.2% collagenase P (Life Technology Corporation) at 37 °C for 30 min. Finally, the 100 µm cell strainer was used to disintegrate the adipose tissue. After centrifuging the sample at 1500 g for 10 min, the cells were seeded into T75 flasks with a density of 1.5*10^6^, and incubated at 37 °C in an incubator with 5% CO_2_. hAD-MSCs could be cultured up to 15–20th passages. hAD-MSCs could be used at 4–5th passages. Specific components of culture medium were described in a previous report [22]. Immunophenotyping of hAD-MSCs was detected in the manuscript of our laboratory [22].

### Extraction of exosomes from HCT116(H-exos)

DMEM/F-12 medium (Life Technology Corporation) was used instead of hAD-MSC culture medium including FBS, and after 36 h, we collected the supernatant and centrifuged it at 3000 g for 30 min. The supernatant was added to an ultrafiltration tube with a 100,000 molecular weight ultrafiltration membrane (Life Technology Corporation). Exosomes were washed twice using D-Hanks’ buffer, and the sample was filtered through a 0.2 µm pore-size membrane, aliquoted and preserved at − 80 °C.

### Exosome uptake

A solution comprising 1 µM 1,1-dioctadecyl-3,3,3,3-tetramethyl indotricarbo- cyanine iodide (DIR) (Life Technology Corporation) was added to the purified exosomes and incubated for 30 min at room temperature. Subsequently, exosomes were added to the MSC culture medium for 6 h. Next, the supernatant was discarded, the cells were washed twice using phosphate buffered saline (PBS), and then Hoechst 33,342 was added to stain the nuclei (1:1000 dilution) for 10 min at room temperature. The images were obtained using a conventional fluorescence microscope (OLYMPUS).

### Clonogenicity assay

First, 1 × 10^3^ MSCs were seeded into each well of a 6-well plate (Corning) containing MSC medium with or without or exosomes, PYR3 (inhibitor of TRPC3) or OAG (activator of TRPC3). After 12 days, the colonies were fixed with 4% paraformaldehyde for 10 min, and then stained with (0.5% w/v) crystal violet for 40 min. The samples were gently washed with sterile PBS and photographed under a microscope.

### Cell–cell co-culture system

The co-culture of 4 × 10^5^ HCT116 cells with the 4 × 10^5^ hAD-MSCs at a 1:1 ratio was in the Transwell^®^ chamber (0.4 µm) (Corning). MSCs were seeded into the upper insert, while HCT116 cells were placed in the lower chamber. Both MSCs and HCT116 Cells were passaged when at 90% confluence. Samples were collected at days 0, 3, 5, 7, and 9 for further analyses.

### siRNAs transfection and virus infection

The siRNAs that were used to knock down TRPC3 mRNA expression (Additional file 1: Table S1) were synthesized by GenePharma company (China). The procedure for virus infection was described in a previous report [23].

### Cell proliferation assay (MTS)

MT-CAFs were pretreated following stimulation with H-Exos(100ug/ml), the TRPC3 agonist OAG(100 µM) and the TRPC3 inhibitor PYR3(6 µM) for 48 h. The specific procedures were the same as described in a previous report [24].

### Cell counting Kit-8 (CCK8)

The CCK-8(JD226-500 T, BEIJING JUDEANTAI TECHNOLOGY CO, LTD) assay was used to detect MT-CAF proliferation in the presence of either PBS, exosomes, exosomes + PYR3 or OAG, respectively. The cells were cultured in 96-well plates with five replicate wells for each treatment. After the cells were cultured for 0, 1, 2, 3 and 4 days, 10 µl CCK-8 solution was added to each well and incubated for 1 h at 37 °C in the cell-culture incubator with 5% CO_2_. Finally, the absorbance of each well was measured at 450 nm.

### Enzyme-linked immunosorbent assay (ELISA)

IL-6 and IL-8 ELISA kit were purchased from Jiangsu Meimian industrial Co., Ltd, China. Cells were cultured for 3 and 7 days in different treatments and the supernatant was then collected to measure the IL-6 and IL-8 levels according to the manufacturer’s instruction.

### Western blot analysis

Western blotting was done as described previously [24]. The TRPC3 (sc-514670, 1:1000) were purchased from Santa Cruz Biotechnology (Delaware, CA, USA); IL-6 (12,912, 1:1000), AKT (9272,1:1000), ERK (4695,1:1000), p-ERK1/2 (4370, 1:1000), p-SMAD3(9520, 1:1000), NF-KB(8242, 1:1000), p-NF-KB (3033, 1:1000) anti-rabbit IgG-HRP (14,708,1:2000), and anti-mouse IgG-HRP (14,709,1:2000) antibodies were obtained from Cell Signaling Technology (Danvers, MA, USA); IL-8 (500-M08, 1:1000), SMAD3 (25,494-1-AP, 1:1000) and p-Akt (ser473, 66,444-1-IG,1:2000) antibodies were purchased from Proteintech (Chicago, IL, USA).

### Cell invasion and migration assay

Colorectal cancer cell migration and invasion was assayed using 24-well Matrigel-coated Transwell inserts (BD Biosciences, San Diego, CA, USA). Invasion and migration of MT-CAFs and HCT116 cells was assayed using previously reported procedures [24].

### Immunohistochemistry (IHC) staining

The tumor tissue samples were fixed with 4% formaldehyde, embedded in paraffin, and sliced into 4 µm thick sections. The samples were further de-waxed and hydrated. Intrinsic peroxidases were inactivated with 3% H_2_O_2_, and the nonspecific protein-binding sites were blocked by adding 10% normal goat serum. The blocked slides with incubated with the primary antibody (1:50) overnight at 4 °C. Then, the secondary antibody was diluted 1:100 in sterile PBS and contacted with the slides at room temperature for 2 h. Finally, hematoxylin was used to counter-stain all immunostained sections. Two pathologists who were blind to the patients’ information evaluated the results of IHC independently. If the result was inconsistent, it was judged by a third pathologist. TRPC3 positivity was defined as IHC staining of > 5% of the cells.

### Immunofluorescence (IF) staining

The cultured cells were washed twice with PBS for 5 min, fixed in 4% formaldehyde for 20 min at room temperature, and washed three times with PBS. Then, 0.1% Triton X-100 in PBS was used to permeabilize the cells for 10 min. Nonspecific binding was blocked with 10% normal goat serum for 30 min. The samples were incubated with the primary antibody (NF-κB, 1:50) at 4 °C overnight, followed by incubation with the secondary Alexa Fluor 488 goat anti-rabbit IgG at room temperature for 1 h. The samples were washed with PBS, and the nuclei counterstained with Hoechst 33,342 (Gibco).

Fluorescent double staining (ACTA2/TRPC3) was done similarly to the immunohistochemistry (IHC) staining, but the secondary Alexa Fluor 488 goat anti-rabbit IgG and Alexa Fluor PE goat anti-rabbit IgG antibodies were contacted with the samples at room temperature for 1 h. The results were observed by confocal laser scanning microscopy (Olympus).

### Xenograft assay in nude mice

The nude mice were purchased from Beijing Vital River Laboratory Animal Technology Co., Ltd. For the subcutaneous tumor model, nude mice were divided into three groups (*n* = 7). One was only injected with 5 × 10^6^ HCT116 cells, one group was co-injected with 1 × 10^6^ MSCs and 5 × 10^6^ HCT116 cells, and the third group was co-injected with 1 × 10^6^ MSCs with TRPC3 knockdown and 5 × 10^6^ HCT116 cells. The specific methods were reported in the literature [24].

### Agilent expression profiling gene chip

Total RNA was extracted using Trizol from MSC cells cultured alone (0 day) and co-cultured with HCT116 cells for the indicated times (1, 3, 5, 7, and 10 days). Sample processing and procedures were done as described in a previous article [24].

### Clinical specimens

After obtaining informed consent, colon cancer specimens were obtained from 63 patients in PUMCH between January 2014 and December 2016. All patients received complete resection and pathologically diagnosed as stage I-III colon cancer. All the protocols were approved by the Ethics Committee of PUMCH.

### Written informed consent

All participants provided written informed consent to take part in the study.

### Statistical analysis

All data are expressed as means ± SD from at least three independent experiments, and statistical analysis was performed using two-tailed Student’s *t*-test and one-way ANOVA. The relationship between TRPC3 expression and disease-free survival (DFS) time was tested using the Kaplan–Meier method. Differences with *P*-values < 0.05 were considered statistically significant. TRPC3 expression.

## Results

### Time series analysis of MSCs treated with H-exos at different time points

**Fig. 1:**
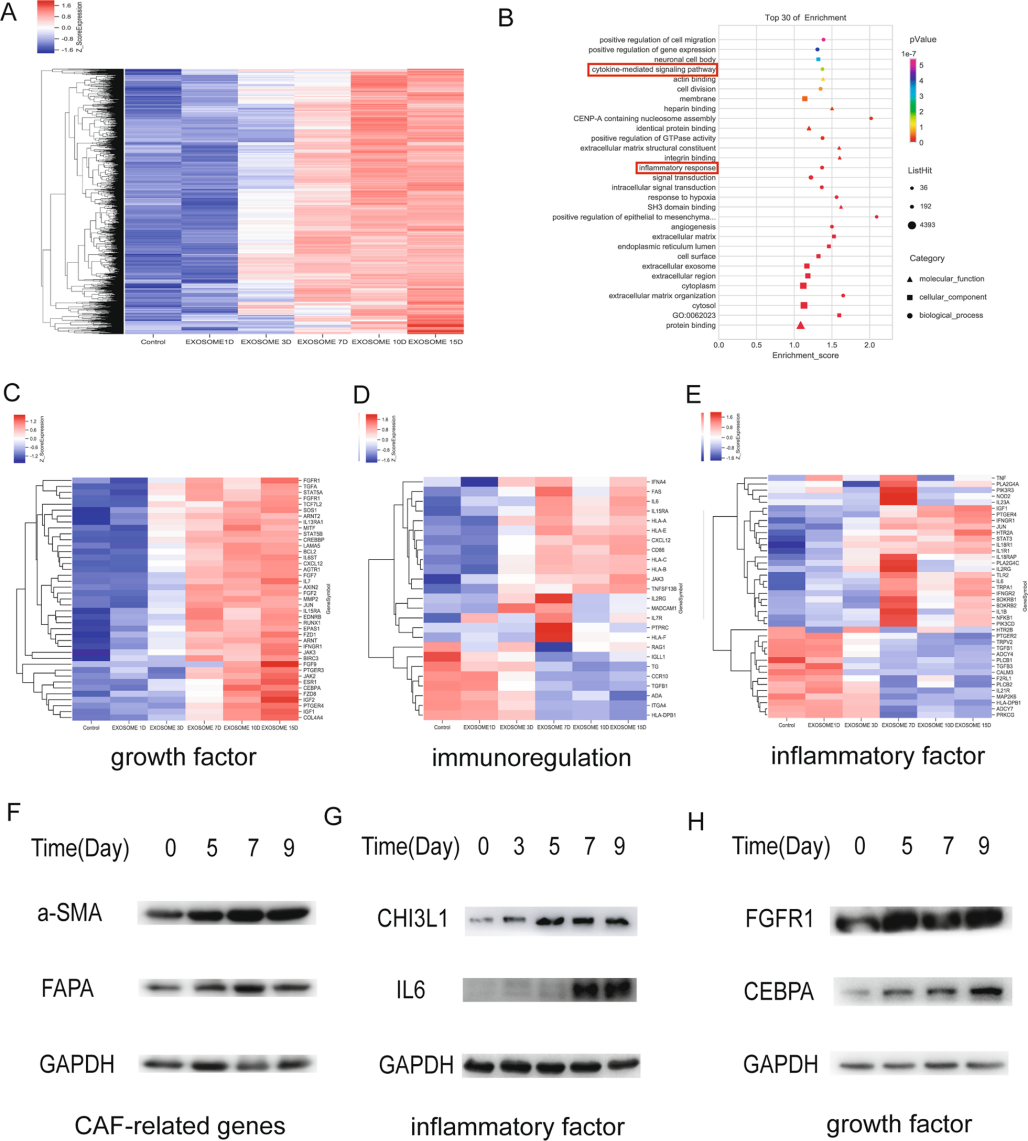
Gene expression profile chip analysis of MSCs treated with H-exos at different time points. **A** Heat map indicated differentially expressed genes between MSCs treated with H-exos and control MSCs. **B**. GO analysis showing the various changes of functions in the MT-CAFs after H-exos stimulation. **C** Genes related to growth factors. **D** Genes associated with immunoregulation. **E** Genes associated with inflammatory factors. **F** WB confirmed the increased expression of related genes associated with CAFs (a-SMA and FAPA). **G** The expression of inflammatory factors was detected by WB (IL6 and CHI3L1). **H** The expression of growth factors was detected by WB (FGFR1 and CEBPA)

The characterization of exosomes derived from H-Exos is summarized in Additional file 2: Fig. S1. We detected changes in gene expression profiles of MSCs after H-Exos stimulation by time series analysis (Fig. 1A). In addition, gene ontology (GO) analysis indicated that there are various changes of functions after exosome stimulation, including inflammatory response, cytokine-mediated signaling pathways and so on (Fig. 1B). Heatmap analysis indicated that upregulation of gene markers was related to growth factors, immune regulation and inflammatory factors in MT-CAFs (Fig. 1C–E). In addition, western blot analysis showed that the expression of CAF-related genes (a-SMA and FAPA) and pro-inflammatory factors (IL6 and CHI3L1) was obviously increased after exosome induction (Fig. 1F, G). The results of western blot analysis also showed that the expression of growth factors (FGFR1 and CEBPA) increased after adding exosome after day 5 (Fig. 1H). Overall, these results indicate that H-Exos might play a critical role in the process of MSC differentiation into the MT-CAFs.

### H-Exos induced the differentiation of MSCs into MT-CAFs by activating the TRPC3 ion channel

**Fig. 2:**
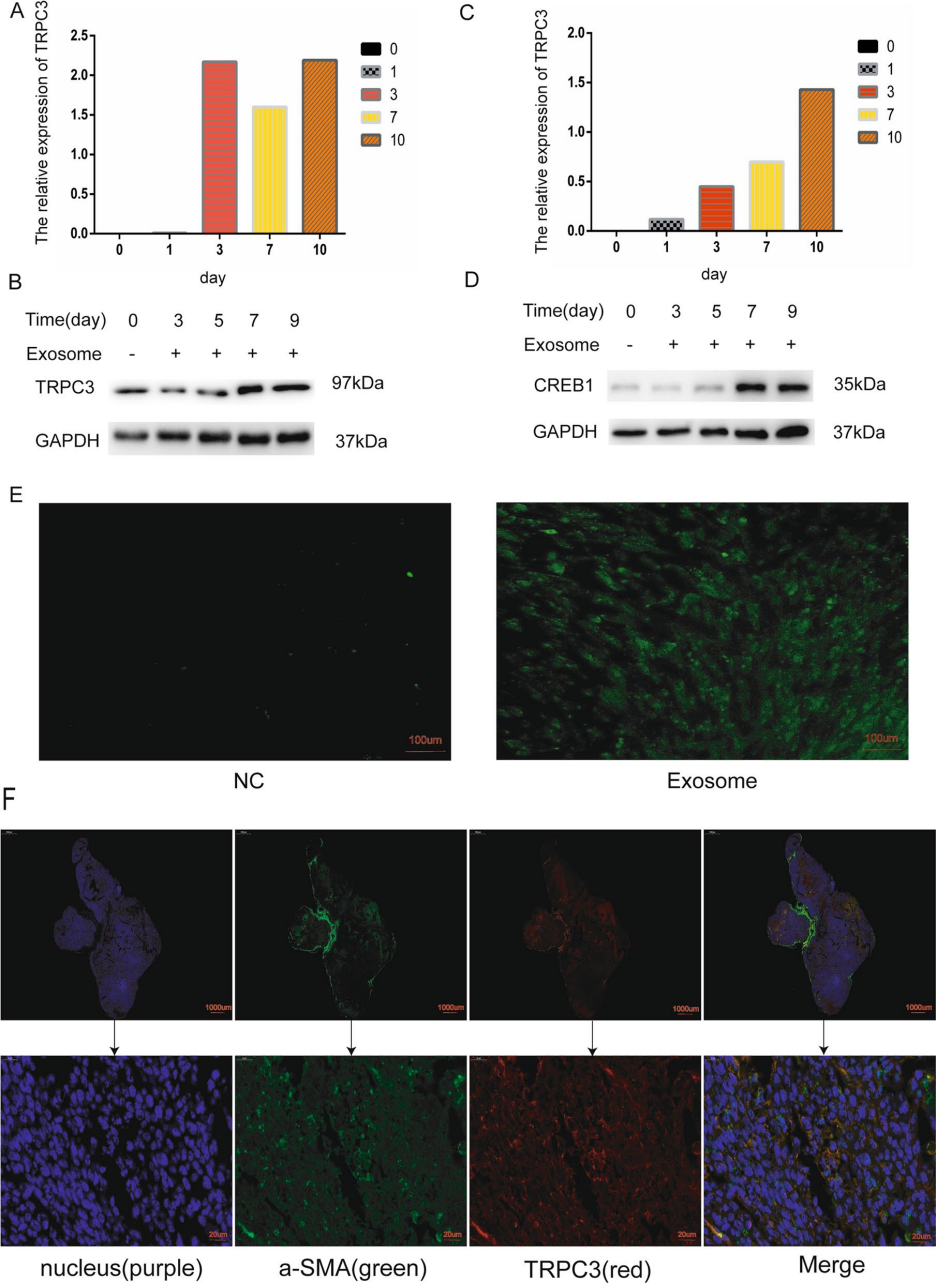
H-exos mediate the differentiation of MSCs into MT-CAFs by activating TRPC3. **A**, **B** The expression of TRPC3 was detected using time series analysis at days 0, 1, 3, 7, and 10, as well as WB at days 0, 3, 5, 7, and 9. **C**, **D** Time-series analysis and WB evaluation of CREB1 expression at days 0, 1, 3, 7, and 10. **E** The cellular Ca^2+^ levels were examined by Furo-4 staining at day 5, the fluorescence intensity was obviously enhanced after adding H-exos (Green). **F** The expression of TRPC3 and a-SMA was detected by immunofluorescence double staining (TRPC3: red; a-SMA: green)

There is increasing evidence that many long-known and newly discovered ion channels including, the Na^+^, K^+^, Ca^2+^ and Cl^−^ channels, may promote the migration and invasion of various cancers [25]. According to the KEGG analysis conducted in this study, the calcium signaling pathway is critical for the differentiation of MSCs into MT-CAFs (Additional file 3: Fig. S2). On this basis, we screened out the TRPC3 mRNA, which exhibited significantly elevated expression after day 3, using time series analysis (Fig. 2A). We further verified that the expression of TRPC3 was increased (Fig. 2B). In addition, the expression of the calcium-related CREB1 protein was obviously improved after exosome stimulation according to both time series analysis (Fig. 2C) and western blot analysis (Fig. 2D). In accordance with the function of TRPC3 as a Ca^2+^ channel, we observed the cellular Ca^2+^ density using the membrane-permeable fluorescent probe Fura-4. The results showed that the cellular Ca^2+^ concentration was obviously increased at day 5 (Fig. 2E). In order to confirm the expression of TRPC3 in the mesenchymal cells from tumor tissue samples, we stained pathological sections of colon cancer tissue from mice. The results showed that a-SMA and TRPC3 were co-expressed, which suggested that TRPC3 was expressed on the surface of the mesenchymal cells in the mouse tumor tissues (Fig. 2F). Thus, the in vitro and in vivo results suggested that the differentiation of MSCs into MT-CAFs is correlated with high expression of TRPC3.

### TRPC3 expressed on MSCs mediates the migration and invasion of MT-CAFs

**Fig. 3:**
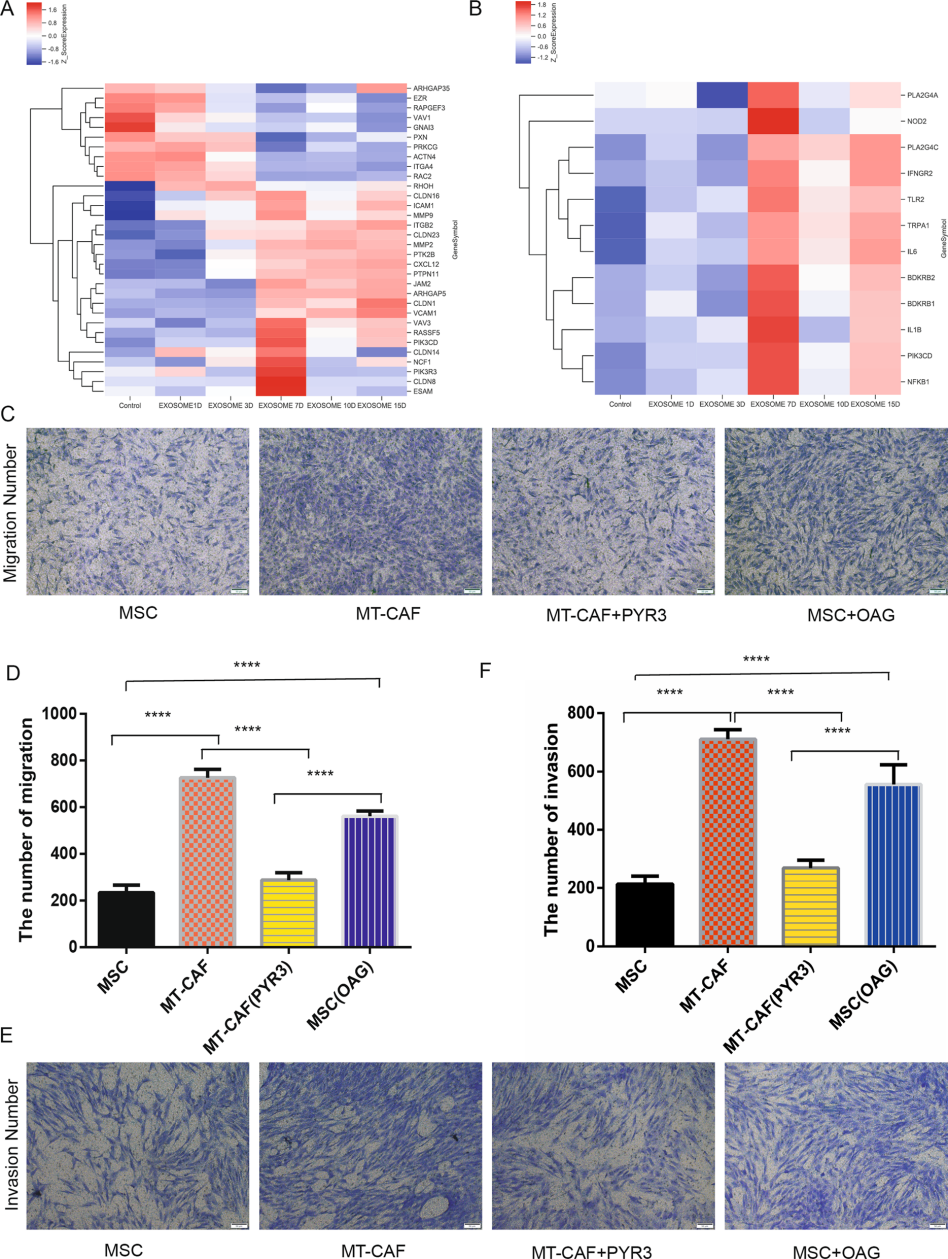
TRPC3 mediates the invasion and migration of MT-CAFs in vitro. **A** Heatmap analysis of the genes related to migration at different time points. **B** Heatmap analysis of the genes related to invasion at different time points. **C** Transwell migration assays showing the migration ability of MT-CAFs in four different groups. **D** Statistical analysis of the results shown in figure **C** (*P* < 0.05). **E** The invasion ability of MT-CAFs was detected using transwell migration assays in four different groups. **F** Statistical analysis of the results shown in figure **E** (*P* < 0.05)

Gene expression profile chip analysis revealed that the invasion and migration-related genes of MT-CAFs were upregulated after H-exos stimulation (Fig. 3A, B). To further verify the function of TRPC3 in the invasion and migration process of MT-CAFs, we detected the invasion and migration of MT-CAFs after adding exosomes alone, with the TRPC3 inhibitor PYR3, or only adding the TRPC3 agonist OAG. The results showed that inhibiting TRPC3 obviously reduced the migration (Fig. 3C, D) and invasion (Fig. 3E, F) of MT-CAFs. Conversely, the TRPC3 agonist OAG significantly increased the migration ability of MSCs, similar to H-Exos stimulation (Fig. 3C, D). The same results were seen when HCT116 cells were co-cultured with MSCs following TRPC3 knockdown, which decreased the migration and invasion ability compared with MT-CAFs (Additional file 4: Fig. S3). These results indicate that TRPC3 expressed on MT-CAFS is involved in their invasion and migration behavior.

### TRPC3 expressed on MT-CAFs contributes to their ability to increase the migration and invasion of HCT116 cells

**Fig. 4:**
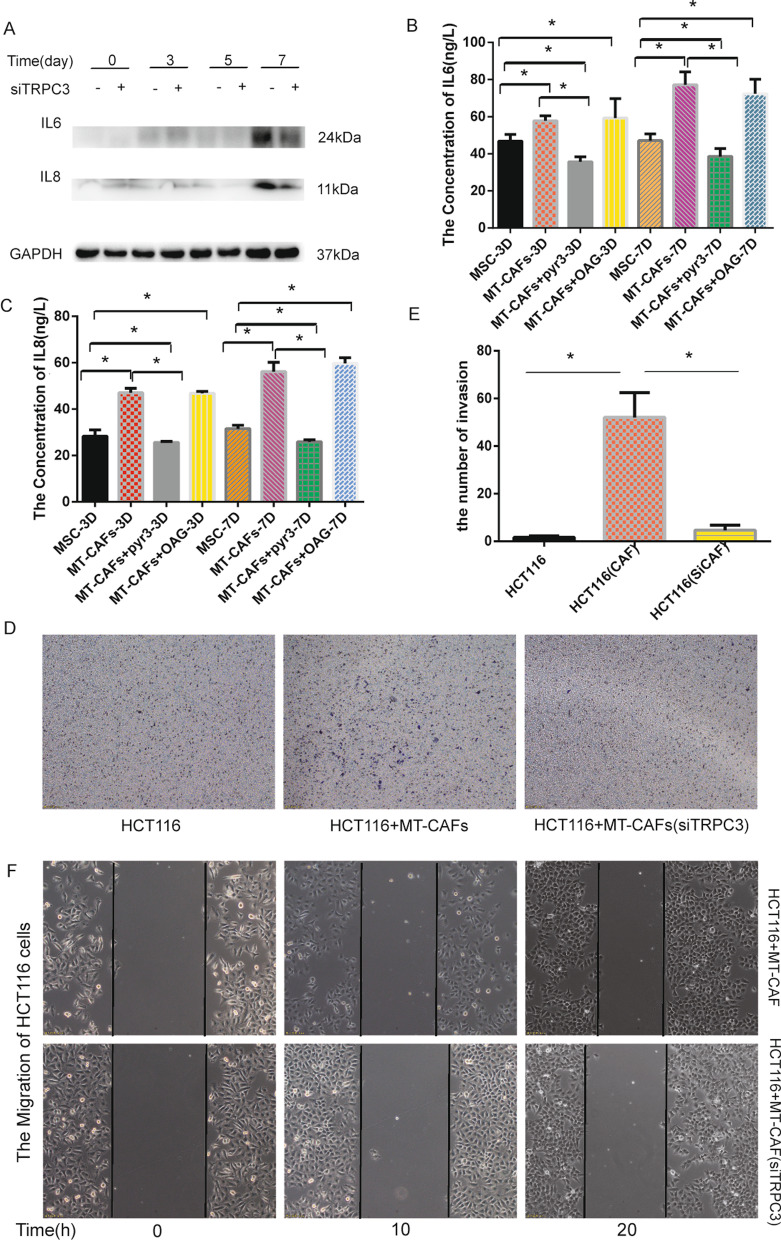
TRPC3 expressed on the MT-MSCs regulates the invasion and migration of colon cancer cells in vitro. **A** The expression of IL6 and IL8 following knockdown of TRPC3 in MT-CAFs and HCT116 cells at different days. **B**, **C** The expression of IL6 and IL8 were detected by ELISA. **D** Transwell migration assays were conducted to evaluate the invasion capacity of HCT116 cells. **E** Quantitative results (*P* < 0.05). **F** The migration ability of HCT116 cells was detected using a wound healing assay

In order to prove the function of TRPC3 expressed on the MT-CAFs in the tumor microenvironment, we detected the expression of the pro-inflammatory factors IL6 and IL8 in MT-CAFs, and the results showed that IL6 and IL8 expression was obviously decreased after knocking down TRPC3 derived from MT-CAFs (Fig. 4A). We collected cell supernatants from three and seven days of different treatment conditions including MSCs alone, exosomes-treated MSCs, exosomes and PYR3 co-treated MSCs, and OAG-treated MSCs. We found that the expression of IL6 and IL8 was significantly increased in the exosomes treatment group compared with the MSCs alone group, and the expression level was significantly down-regulated in the exosomes and PYR3 co-treatment group, but significantly reversed this phenomenon in the OAG treatment group(Fig. 4B, C). We next analyzed whether MT-CAFs with TRPC3 knockdown affected tumor metastasis by co-culturing HCT116 cells with MT-CAFs with or without TRPC3 knockdown. We found that knocking down the TRPC3 ion channel obviously attenuated the invasion (Fig. 4D, E) and migration (Fig. 4F) ability of HCT116 cells. These results indicate that TRPC3 expressed on MT-CAFs plays a role in the promotion of tumor cell invasion and migration by MSCs.

### TRPC3 enhanced the proliferation and colony formation of MT-CAFs

**Fig. 5:**
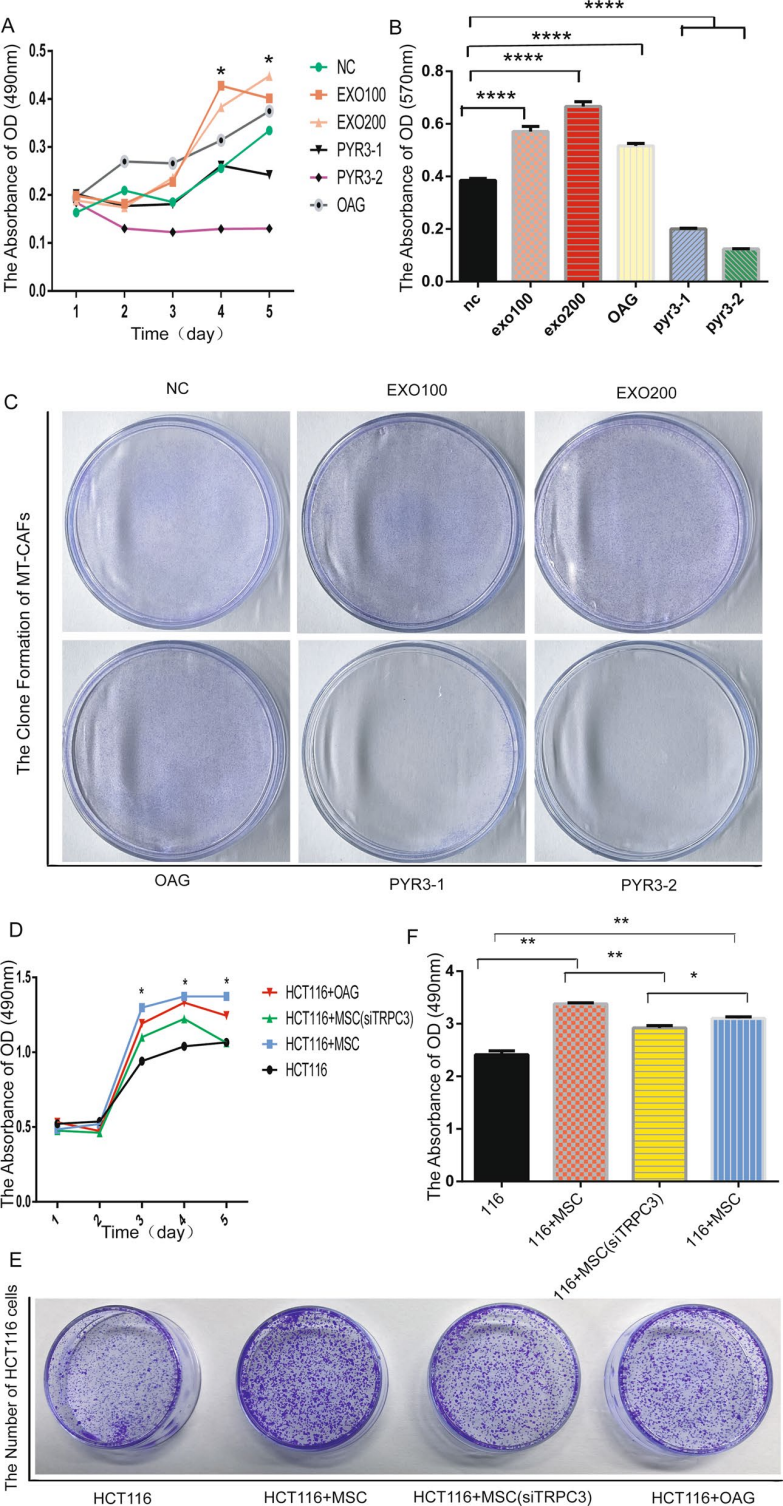
TRPC3 regulates the proliferation of MT-MSCs in vitro. **A** MTS assay in MT-CAFs with different treatments including control MSCs, MSCs with 100 ng or 200 ng of stimulus, MSCs with OAG (TRPC3 agonist), MSCs with pyr3 (TRPC3 inhibitor 1 µM or 2 µM). The data represent the means ± S.D. (*P < 0.05; **P < 0.01 by Student’s *t*-test). **B** Clone formation assay in MT-CAFs with six different treatments. **C** Quantitative results (*P* < 0.05). **D** MTS assay in HCT116 cells following different treatments. **E** Clone formation assay in HCT116 cells with four different treatments. **F** Statistical analysis of the results shown in figure **E** (*P* < 0.05)

To further verify the impacts of TRPC3 on the proliferation of MT-CAFs, MTS assays were performed. The growth of MT-CAFs was obviously enhanced following stimulation with H-Exos or the TRPC3 agonist OAG. Conversely, the growth of MT-CAFs was significantly decreased by the addition of the TRPC3 inhibitor PYR3 (Fig. 5A). In addition, the colony formation assay showed that the number of clones was increased following the activation of TRPC3, while it was greatly decreased after inhibition of TRPC3 in MT-CAFs (Fig. 5B, C). These results suggested that TRPC3 plays an important role in regulating cell proliferation and colony formation of MT-CAFs.

### TRPC3 expressed on MSCs mediates the invasion and migration of MT-CAFs by activating NF-KB signaling pathway

**Fig. 6:**
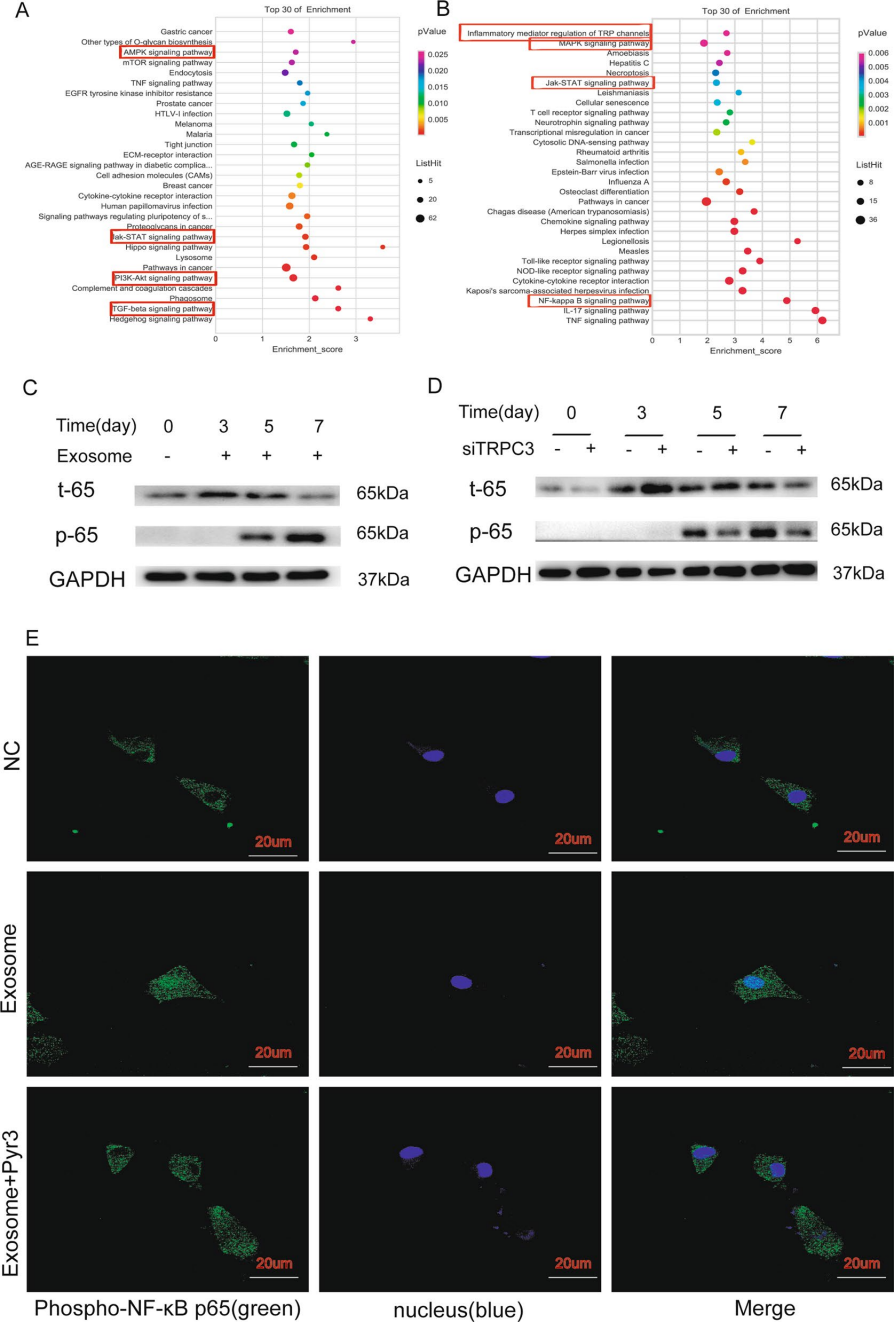
H-exos regulate the function of MT-CAFs via the TRPC3/NF-κB signaling pathway. **A**, **B** Analysis of gene expression profiles indicated that various signaling pathways were activated in MT-CAFs following stimulation with H-exos. **C** WB confirmed that the relevant signaling pathways were activated in MT-CAFs compared to the MSCs. **D** Activation of NF-κB was positively correlated with TRPC3 in MT-CAFs according to western blot analysis. **E** The phosphorylation of NF-κB protein were detected by immunofluorescence staining

Analysis of gene expression profiles showed that many signaling pathways were activated in MT-CAFs. Among them, we selected the PI3K-Akt, JAK-STAT, AMPK, TGFβ, MAPK and NF-κB signal pathways for further analysis (Fig. 6A, B). Western blot analysis confirmed that the phosphorylation of NF-κB plays a critical role in the differentiation of MSCs into MT-CAFs after H-Exos stimulation (Fig. 6C). Furthermore, phosphorylation of NF-κB had a positive relationship with the TRPC3 ion channel. Accordingly, knocking down TRPC3 significantly inhibited the phosphorylation of NF-κB in the MT-CAFs after day 5 (Fig. 6D). However, none of the other investigated signaling pathways exhibited significant changes following TRPC3 knockdown in MT-CAFs (Additional file 5: Fig. S4). To further investigate the phosphorylation of NF-κB in MT-CAFs induced by the TRPC3 ion channel, we detected the phosphorylation of NF-κB in cells with or without PYR3 treatment (1.0 μM for 72 h). The phosphorylation and nuclear transport of NF-κB was obviously decreased according to immunofluorescence staining (Fig. 6E). These results suggested that H-Exos can activate the NF-κB signaling pathway in MT-CAFs via the TRPC3 ion channel, which may regulate the biological function of these cells.

### TRPC3 expressed on MT-CAFs promoted the tumorigenesis and progression of xenografts in nude mice

**Fig. 7:**
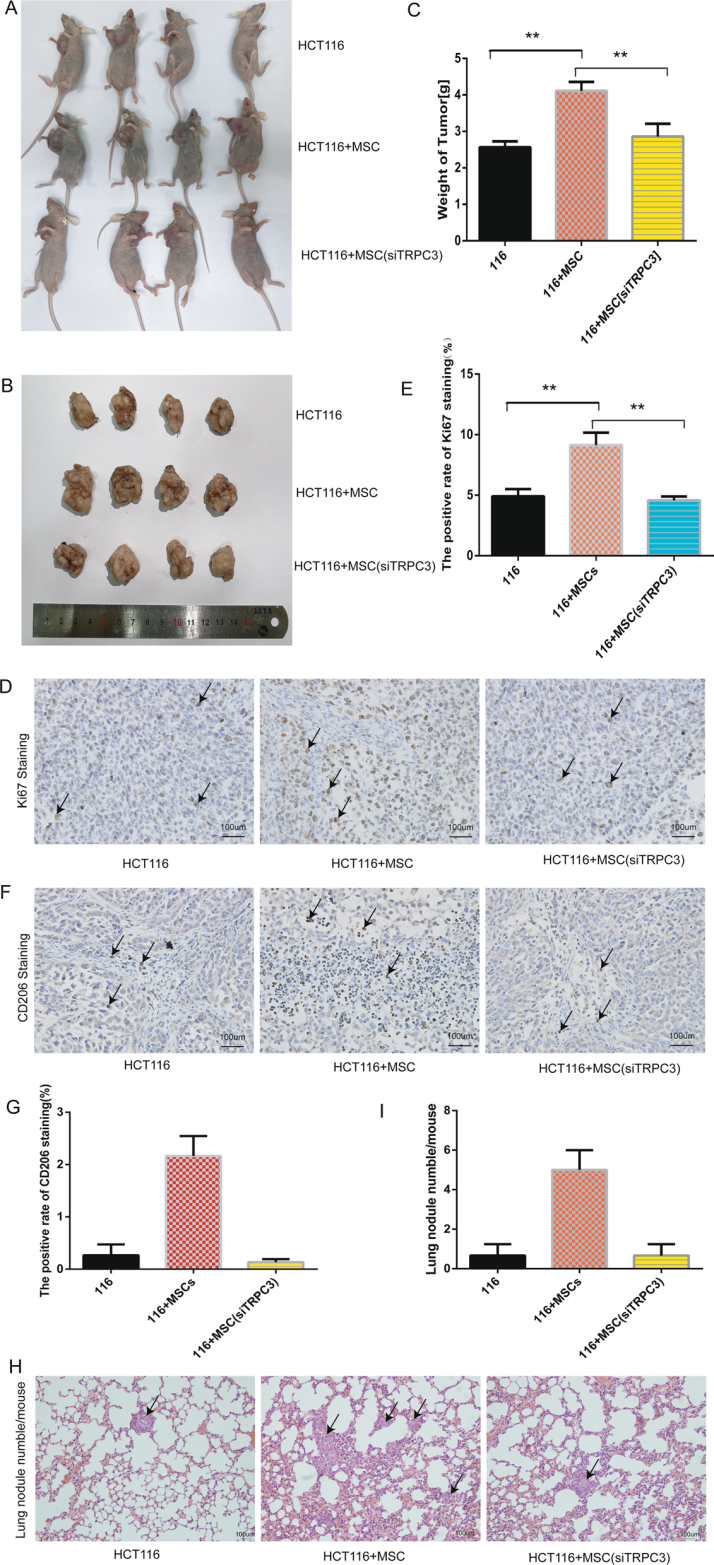
TRPC3 expressed on the MT-CAFs contributes to the growth and progression of colorectal cancer xenografts in vivo. **A**/**B** Representative photographs of HCT116 tumors generated in the three different groups. **C** Quantitative analysis of the results shown in figure B (*P* < 0.05). **D**/**E** The expression of proliferation factor ki67 in tumor tissues of different groups was assessed by IHC, figure E shows the quantitative data (*P* < 0.05). **F**/**G** Macrophage infiltration into tumor tissues was detected by staining for CD206 using immunohistochemistry. Representative images of CD206 staining for each group are shown (magnification × 200). **H** The number of lung metastases was examined by HE staining. **I** Quantitative analysis of the results shown in figure H (*P* < 0.05)

To further verify the pathological role of TRPC3 in vivo, we constructed a xenograft tumor model by subcutaneously injecting cells into nude mice. The first group was injected with only 5 × 10^6^ HCT116 cells (H), the second was co-injected with 5 × 10^6^ HCT116 cells and 1 × 10^6^ MSCs (HM), and the third group was co-injected 5 × 10^6^ HCT116 cells and 1 × 10^6^ MSCs with TRPC3 knockdown (HMK). The tumors in the H group were smaller than in the HM group after 6 weeks (*n* = 7). Furthermore, the mean size of tumor in the HMK group was significantly smaller than in the HM group (Fig. 7A, B). In addition, we measured the weight of the tumors after sacrificing the mice, and the tumors in the H and HM groups were significantly lighter than the tumors from the HMK group (Fig. 7C). Additionally, the ki67 positive rate in tumor tissues of different groups was detected by IHC to assess the tumor proliferation in vivo. The results indicated that the rate of proliferation in the HM group was significantly higher than in the other two groups (Fig. 7D, E). It has been reported that tumor-related macrophages (M2 macrophages) can contribute to tumor growth. In this study, CD206^+^ macrophages were significantly more abundant in the tumor tissue samples of the HM group compared to the H and HM groups (Fig. 7F, G). To further investigate the role of TRPC3 in metastasis, we assessed the presence of metastatic nodules in the lungs of these three groups of mice. The number of lung nodules was much higher in the HM group than in the H and HMK groups (Fig. 7H, I). Taken together, these results indicate that TRPC3 expressed on the MT-CAFs contributes to the growth and metastasis of tumors in vivo.

### TRPC3 expression in MT-CAFs is correlated with poor prognosis in colorectal cancer patients

**Fig. 8:**
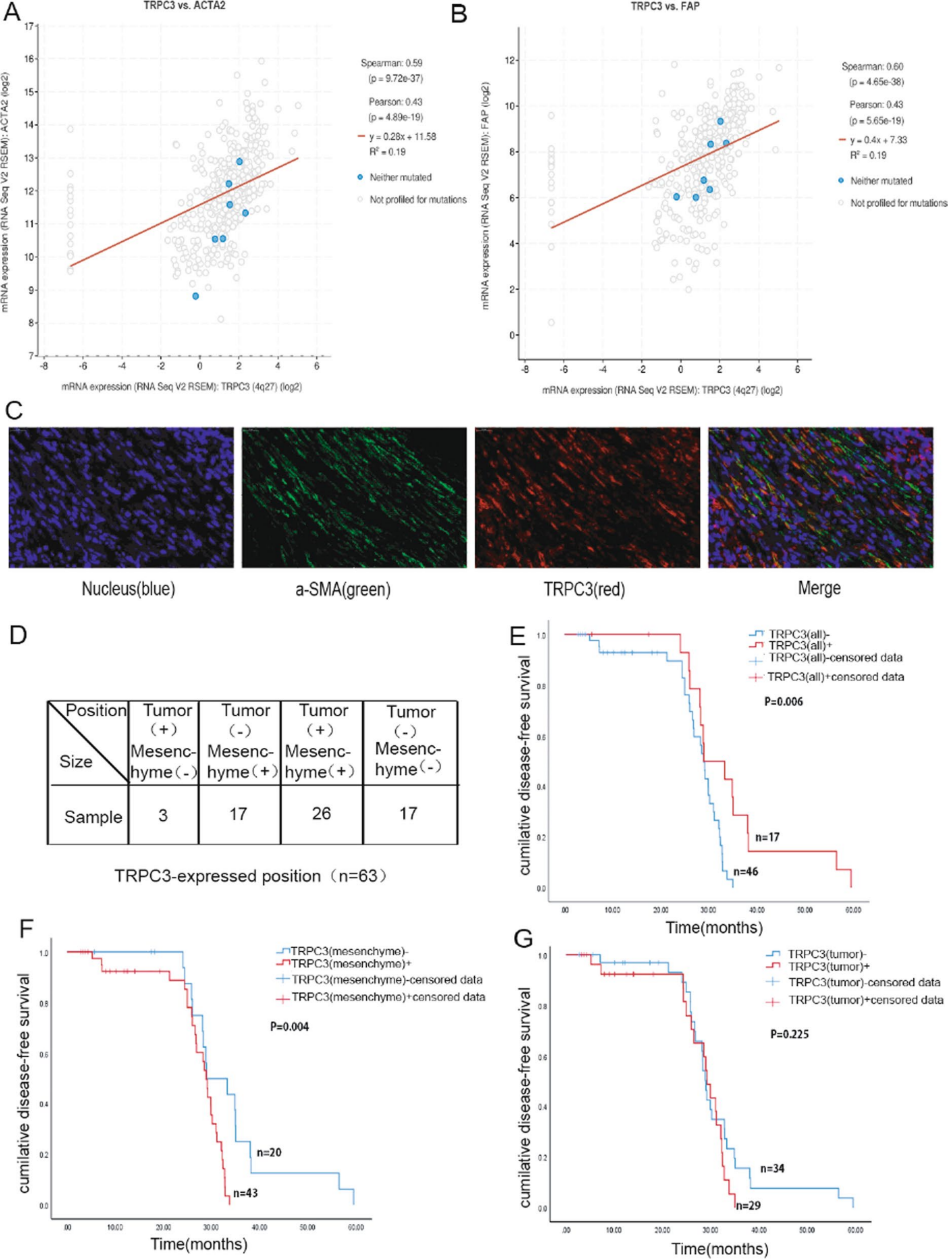
TRPC3 expression was related to a poor prognosis in patients. **A**/**B** The correlation of TRPC3, ACTA2 and FAP was analyzed using the cBioPortal database (*P* < 0.05). **C** Colon cancer tissues were stained by immunofluorescence using antibodies against TRPC3 (red) and a-SMA (green); the nuclei were counterstained with Hoechst 33,342 (blue). **D** A total of 63 patients were divided into four groups, including only mesenchymal TRPC3 positive, tumoral TRPC3 positive, double positive, and double negative. **E**/**F**/**G** Kaplan–Meier curves for DFS stratified by overall TRPC3 expression, mesenchymal TRPC3 expression and tumoral TRPC3 expression

To explore the relationship between TRPC3 expression and the prognosis of colorectal cancer patients, we acquired relevant data from the public Oncomine database (www.oncomine.org), which includes colon tissues and colorectal cancer tissues. We found that TRPC3 expression in colorectal cancer tissues was higher than in normal colon tissues (Additional file 6: Fig. S5A). Moreover, the expression trend of CAF-related genes (ACTA2 and FAP) was consistent with that of TRPC3 expression (Additional file 6: Fig. S5B, C). Interestingly, the expression of TRPC3 showed a positive correlation with that of ACTA2 and FAP, which indicates that TRPC3 may regulate the function of CAFs (Fig. 8A, B). To further verify the relationship between TRPC3 and CAFs, we detected the expression of TRPC3 and ACTA2 in clinical specimens of colorectal cancer patients. The result showed that CAFs in colorectal cancer tissues co-expressed TRPC3 and ACTA2 (Fig. 8C). Furthermore, we investigated the expression of TRPC3 in samples from 63 colorectal cancer patients by IHC. The results showed that the cancers from 17 patients (26.98%) were TRPC3 positive only in mesenchyme cells (MCs), those from 3 patients (4.76%) were positive only in tumor cells (TCs), and the cancers from 26 patients (41.27%) were positive for TRPC3 expression in both mesenchymal and tumor cells (double positive, DP). Additionally, the cancers from 17 patients (26.98%) did not show any TRPC3 expression (double negative, DN) (Fig. 8D).

We then analyzed the correlation between TRPC3 expression and disease-free survival (DFS) of patients using Kaplan–Meier analysis. The median DFS (mDFS) of patients with mesenchymal and tumor positive TRPC3 expression [27.37 m (95%CI = 25.23–29.51)] was significantly shorter than that of patients with double negative expression [34.69 m (95%CI = 28.99–40.38)] (*P* = 0.006), which indicated that TRPC3 expression in clinical specimens of colorectal cancer patients is related with a poorer prognosis (Fig. 8E). To determine whether TRPC3 in mesenchymal cells or tumor cells was more essential to clinical outcomes, we compared the ratio of tumors with TRPC3 positive mesenchymal cells in the TC and DN groups. There was a positive correlation between TRPC3 expression in mesenchymal cells and mDFS [27.20 (95%CI 24.97–29.43) months vs. 34.06 (28.94–39.18) months, *P* = 0.004] (Fig. 8F). However, there was no significant correlation between TRPC3 expression in tumor cells and mDFS [27.79 (95%CI 24.91–30.67) months vs. 30.99 (27.27–34.72) months, *P* = 0.225] (Fig. 8G). Additionally, patients with positive expression exclusively in MCs and DP had shorter mDFS than patients with DN tumors (*P* = 0.018) (Additional file 7: Fig. S6). These results suggest that the expression of TRPC3 in mesenchymal cells is related with a poor prognosis in colorectal cancer patients. This result is consistent with the experimental findings indicating a role of TRPC3 expression in CAFs in vitro*.*

## Discussion

Increasingly, studies are demonstrating that the tumor microenvironment itself can be considered a therapeutic target for cancer therapy. Particularly, cancer-associated fibroblasts (CAFs) play a crucial role in the progression of tumors. Studies have shown that the major sources of CAFs in pancreatic ductal adenocarcinoma are pancreatic stellate cells and bone marrow-derived mesenchymal stem cells (MSCs) [26, 27]. Importantly, it has been shown that MSCs derived from adipose tissue are another source of CAFs with a high self‐renewal capacity [28, 29]. In this study, we showed that H-Exos can induce the differentiation of MSCs derived adipose tissue into MT-CAFs. The expression of specific markers on CAFs increased with time, suggesting that MT-CAFs were formed. MSCs derived from adipose tissue can be easily obtained. The model of the differentiation of MSCs into CAFs induced by tumor exosomes can be used as a suitable cell model to study the molecular mechanism guiding the development of tumor stromal cells.

In this study, we screened out the functional protein TRPC3 in MT-CAFs using a profile chip to analyze gene expression at different time points. As a Ca^2+^ ion channel, TRPC3 plays a critical role in different tumors by activating distinct signaling pathways. It was reported that the novel TRPC3-RASA4-MAPK signaling pathway mediates the growth, apoptosis and chemoresistance in triple negative breast cancer [19]. In melanoma, the lncRNA SNHG5 was found to affect tumor development by regulating the miR-26a-5p/TRPC3 pathway [20]. In prostate cancer, TRPC3 was found to promote angiogenesis, which plays a critical role in cancer progression [30]. TRPC3 is also a regulator of follicle-stimulating hormone, which could be a potential therapeutic target for ovarian cancer. Accordingly, estrogen enhances the progression of ovarian cancer cells by activating TRPC3 [31]. However, the function of TRPC3 in the CAFs still remains to be elucidated. In this study, we used a gene expression profile chip to detect the changes of various signaling pathways in MT-CAFs. The results revealed that a number of signaling pathways were activated by exosomal stimulation. However, only the NF-κB signaling pathway showed a positive relationship with TRPC3. Furthermore, the knockdown or inhibition of TRPC3 obviously decreased the proliferation and metastasis ability of MT-CAFs. TRPC3 expression in MT-CAFs also affects their ability to promote tumor progression. Inhibition of TRPC3 expression weakened the ability of CAFs to promote tumor cell proliferation, invasion and migration, while a TRPC3 agonist alone was able to promote tumor cell proliferation, invasion and migration. These results suggest that CAF expression of TRPC3 is closely related to the promotion of tumor progression in vitro.

Furthermore, we adopted a xenograft tumor model to verify the function of TRPC3 in CAFs in vivo. Knockdown of TRPC3 in MSCs obviously decreased tumor growth and progression compared to co-culture with HCT116 and MSC cells. This was consistent with the finding that TRPC3 expressed on MT-CAFs has a positive effect on tumor-cell proliferation and metastasis in vitro. We also detected more lung metastases in xenografts composed of HCT116 and MSCs compared to the other two groups. Studies have also shown that M2 macrophages contribute to the growth of cancer cells in multiple malignancies, including gastric and breast cancer. In this study, we found that the abundance of M2 macrophage was obviously increased in the HM group compared to the H and HMK groups. It is therefore possible that MSCs can contribute to the growth of colon cancer by recruiting M2 macrophages. The in vivo results also suggest that TRPC3 is closely related to the ability of CAFs to promote tumor progression.

To further verify the expression of TRPC3 in CAFs from colorectal cancer patients and its correlation with the prognosis, we used IHC to detect the expression of TRPC3 in clinical specimens. Despite the limited number of cases, the data revealed that TRPC3 expression in CAFs was increased in colon cancer tissues at different stages. These results were confirmed using data from the online Oncomine database. The results obtained from the data available for colon cancer in Oncomine showed that the expression of TRPC3 was positively related with the expression of CAF marker genes (α-SMA and FAP). The correlation between the expression of TRPC3 in tumor cells or tumor stromal cells and patient prognosis was further analyzed. The DFS was significantly shortened in patients with TRPC3-positive stromal cells compared to those with TRPC3-negative stromal cells. By contrast, there was no statistically significant difference in DFS between tumoral TRPC3-positive and TRPC3-negative patients. In addition, the DFS was significantly shorter in TRPC3-positive patients (including tumor-positive and stromal cells) than in TRPC3-negative patients. Our results suggest that TRPC3 expression is associated with a poor prognosis, and that the role of TRPC3-positive mesenchymal cells is more important than that of TRPC3-positive tumor cells.

## Conclusions

MSCs derived from adipose tissue can differentiate into MT-CAFs by co-culture with HCT116 cells or H-exos in vitro. Using this induced CAF model, we screened out the TRPC3 gene expressed in CAFs as a biomarker, and confirmed that it influences the invasion and migration ability of cancer cells, promoting the progression of colon cancer by activating the NF-κB signaling pathway. Examination of clinical colorectal cancer specimens confirmed that the expression of TRPC3 in tumors, and especially in CAFs, was closely related with a poor prognosis. This study suggests that MT-CAFs induced by H-Exos can be used as a practical cell model for tumor stroma research. Finally, the results indicate TRPC3 may be a novel prognostic biomarker and a potential therapeutic target for the treatment of colon cancer.

## Supplementary Information

**Additional file 1: Table S1.** Sequences for primers.

**Additional file 2: Fig. S1.** Characterization and uptake of exosomes. **A** H-exos were observed using electron microscopy, which revealed a size range of 30–200 nm. **B** The marker genes of H-exos were detected using WB (HSP70, HSP90, and CD63). **C** NTA analysis to evaluate the size distribution of the H-exos. **D** Immunofluorescence microscopy was sued to detect the uptake of DiR-labeled H-exos by MSCs (Red, 1:1000 dilution), cell nuclei were counterstained with Hoechst 33342 (1:1000 dilution).

**Additional file 3: Fig. S2.** The calcium signaling pathway plays a critical role in the development of MT-CAFs KEGG analysis indicated that the calcium signaling pathway is critical for the differentiation of MSCs into MT-CAFs.

**Additional file 4: Fig. S3.** TRPC3 expressed on MT-CAFs contributes to the metastasis of MT-CAFs. **A** Transwell migration assays were conducted to evaluate the migration capacity of MT-CAFs. **B** Quantitative results (*P* < 0.05). **C** Transwell invasion assays showing the invasion ability of MT-CAFs in three different groups. **D** Quantitative analysis of the results shown in figure C (*P* < 0.05).

**Additional file 5: Fig. S4.** H-Exos can activate different signaling pathways in MT-CAFs. **A** WB showed that different signaling pathways were activated in MT-CAFs by H-Exos stimulation. **B** WB showing the expression of different signaling pathways with or without TRPC3 in MT-CAFs at different days.

**Additional file 6: Fig. S5.** TRPC3 was overexpressed in tumor tissues in the Oncomine database. **A**–**C** The expression analysis of TRPC3, FAP and ACTA2 in tumor samples from the Oncomine database (*P* < 0.05).

**Additional file 7: Fig. S6.** TRPC3 is a predictor for poor prognosis in colon cancer patients. **A**/**B**/**C** Kaplan-Meier curves for DFS of patients with tumors showing only mesenchymal TRPC3, double positive TRPC3, and only tumoral TRPC3 expression.

## Data Availability

All data generated or analyzed during this study are included in this published article.

## Abbreviations

MSCs: Mesenchymal stem cells
hADSCs: Human adipose-derived mesenchymal stem cells
CAFs: Cancer-associated fibroblasts
MT-CAF: MSC-transformed CAF
*α*-SMA: Alpha-smooth muscle
FAP: Fibroblast activation protein alpha
CRC: Colorectal cancer
TME: Tumour microenvironment
IL-6: Interleukin-6
DMEM: Dulbecco’s modified eagle medium
TEM: Transmission electron microscopy
IHC: Immunohistochemistry
IF: Immunofluorescence
PUMCH: Peking union medical college hospital
H-exos: Exosomes from HCT116
DIR: 1,1-Dioctadecyl-3,3,3,3-tetramethyl indotricarbo- cyanine iodide
PBS: Phosphate buffered saline
MTS: Cell proliferation assay
CCK8: Cell counting Kit-8
ELISA: Enzyme-linked immunosorbent assay
IF: Immunofluorescence
DFS: Disease-free survival
