# Supplementary material for: Mesenchymal stem cells derived from adipose accelerate the progression of colon cancer by inducing a MT-CAFs phenotype via TRPC3/NF-KB axis
Source: Stem Cell Res Ther. 2022 Jul 23;13:335. doi: 10.1186/s13287-022-03017-5 (PMC9308187; doi:10.1186/s13287-022-03017-5)
Supplement: Supplementary file 1 — Additional file 1: Table S1. Sequences for primers. [file 13287_2022_3017_MOESM1_ESM.doc]

Table S1 Sequences for primers.

| items | direction | sequence |
| --- | --- | --- |
| IL6 primer | sense | ACTCACCTCTTCAGAACGAATTG |
|  | reverse | CCATCTTTGGAAGGTTCAGGTTG |
| IL8 primer | sense | ACTCCAAACCTTTCCACCCC |
|  | reverse | TTCTCAGCCCTCTTCAAAAACTTC |
| GAPDH primer | forward | GGTCACCAGGGCTGCTTTTA |
|  | reverse | GGATCTCGCTCCTGGAAGATG |
| TRPC3 primer | forward | AGCCAACACGTTATCAGCAG |
|  | reverse | CCAGGTTGCTGCATCATTCAC |
