# Supplementary figures and images for: Mesenchymal stem cells derived from adipose accelerate the progression of colon cancer by inducing a MT-CAFs phenotype via TRPC3/NF-KB axis
Source: Stem Cell Res Ther. 2022 Jul 23;13:335. doi: 10.1186/s13287-022-03017-5 (PMC9308187; doi:10.1186/s13287-022-03017-5)

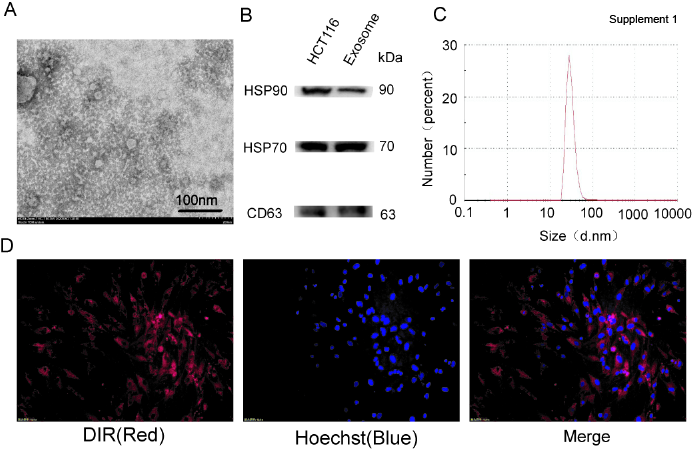

Supplement: Supplementary file 2 — Additional file 2: Fig. S1. Characterization and uptake of exosomes. A H-exos were observed using electron microscopy, which revealed a size range of 30–200 nm. B The marker genes of H-exos were detected using WB (HSP70, HSP90, and CD63). C NTA analysis to evaluate the size distribution of the H-exos. D Immunofluorescence microscopy was sued to detect the uptake of DiR-labeled H-exos by MSCs (Red, 1:1000 dilution), cell nuclei were counterstained with Hoechst 33342 (1:1000 dilution). [file 13287_2022_3017_MOESM2_ESM.tif]

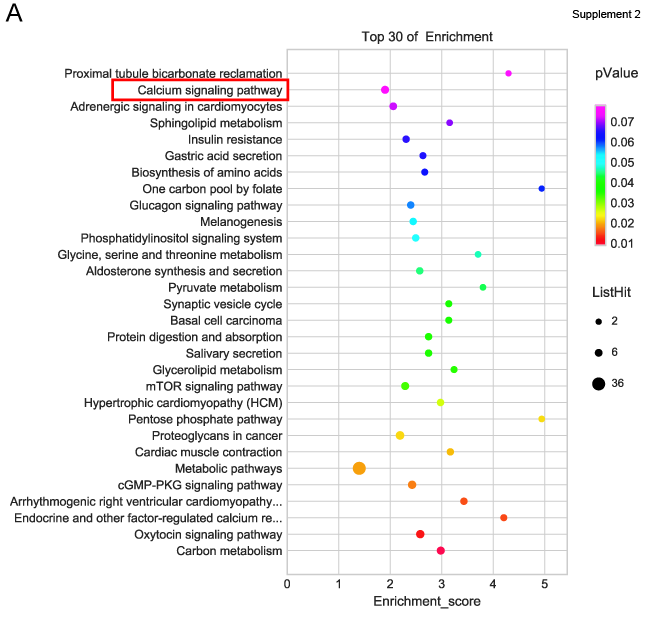

Supplement: Supplementary file 3 — Additional file 3: Fig. S2. The calcium signaling pathway plays a critical role in the development of MT-CAFs KEGG analysis indicated that the calcium signaling pathway is critical for the differentiation of MSCs into MT-CAFs. [file 13287_2022_3017_MOESM3_ESM.tif]

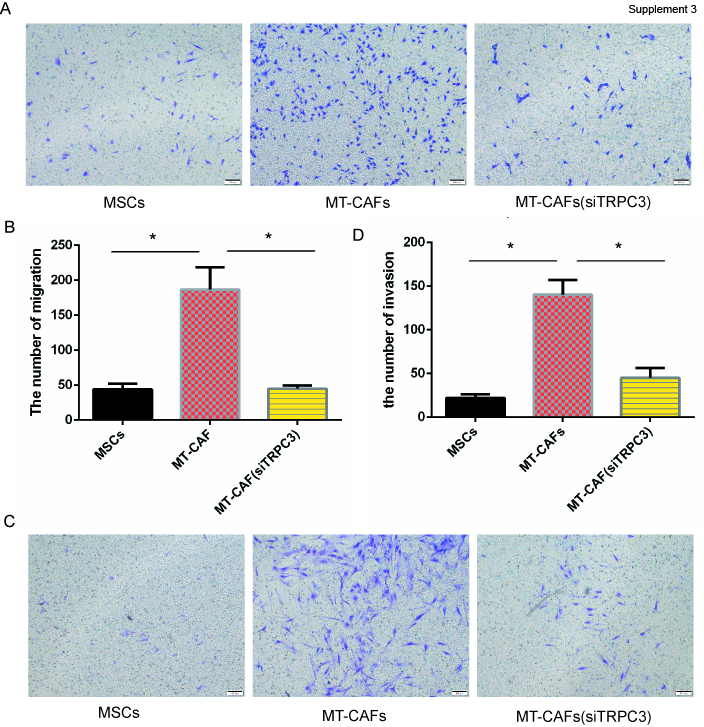

Supplement: Supplementary file 4 — Additional file 4: Fig. S3. TRPC3 expressed on MT-CAFs contributes to the metastasis of MT-CAFs. A Transwell migration assays were conducted to evaluate the migration capacity of MT-CAFs. B Quantitative results (P < 0.05). C Transwell invasion assays showing the invasion ability of MT-CAFs in three different groups. D Quantitative analysis of the results shown in figure C (P < 0.05). [file 13287_2022_3017_MOESM4_ESM.tif]

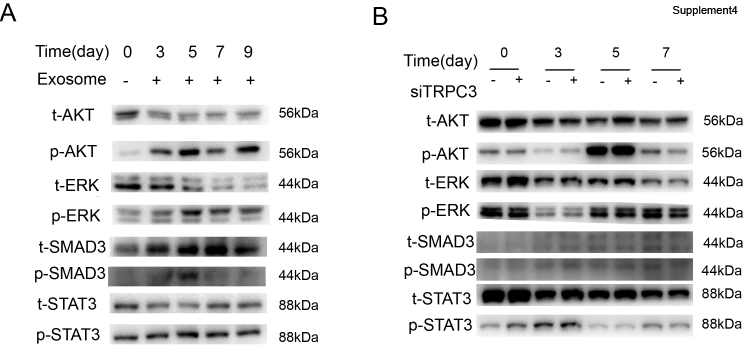

Supplement: Supplementary file 5 — Additional file 5: Fig. S4. H-Exos can activate different signaling pathways in MT-CAFs. A WB showed that different signaling pathways were activated in MT-CAFs by H-Exos stimulation. B WB showing the expression of different signaling pathways with or without TRPC3 in MT-CAFs at different days. [file 13287_2022_3017_MOESM5_ESM.tif]

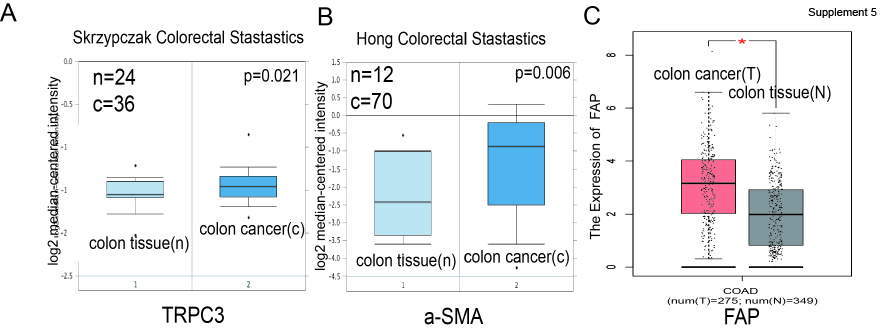

Supplement: Supplementary file 6 — Additional file 6: Fig. S5. TRPC3 was overexpressed in tumor tissues in the Oncomine database. A–C The expression analysis of TRPC3, FAP and ACTA2 in tumor samples from the Oncomine database (P < 0.05). [file 13287_2022_3017_MOESM6_ESM.tif]

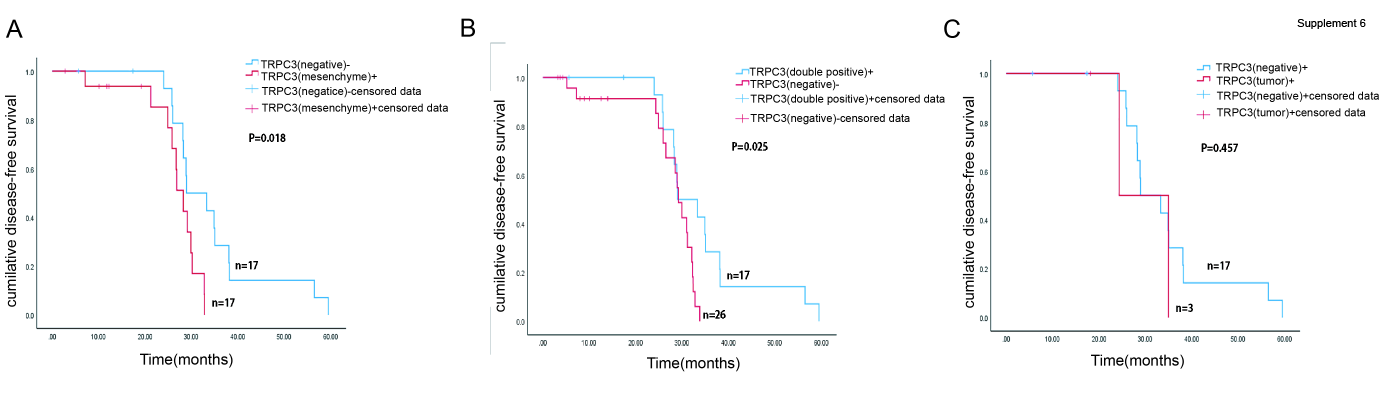

Supplement: Supplementary file 7 — Additional file 7: Fig. S6. TRPC3 is a predictor for poor prognosis in colon cancer patients. A/B/C Kaplan-Meier curves for DFS of patients with tumors showing only mesenchymal TRPC3, double positive TRPC3, and only tumoral TRPC3 expression. [file 13287_2022_3017_MOESM7_ESM.tif]
